# Editorial: The adaptive value of languages: non-linguistic causes of language diversity, volume II

**DOI:** 10.3389/fpsyg.2024.1387290

**Published:** 2024-03-06

**Authors:** Antonio Benítez-Burraco, Steven Moran

**Affiliations:** ^1^Department of Spanish, Linguistics, and Theory of Literature, Faculty of Philology, University of Seville, Seville, Spain; ^2^Department of Biology, University of Neuchâtel, Neuchâtel, Switzerland; ^3^Department of Anthropology, University of Miami, Coral Gables, FL, United States

**Keywords:** language diversity, non-linguistic drivers, typology, adaptation, complexity

After the successful first volume of *The adaptive value of languages: non–linguistic causes of language diversity*, Frontiers asked us to revisit this topic by eliciting and editing a second collection of original research articles. Our goal remains to determine whether linguistic and extralinguistic factors are constrained in systematic ways, which would allow researchers to investigate how non-linguistic factors contributed to, and resulted in, the vast language diversity that we observe today. And more generally, how human language, cognition, and culture interact to account for such diversity. Research in this vein aims at understanding whether some aspects of language structure are due to adaptation from factors including the natural and social environments (e.g., Trudgill, 2011; Nettle, 2012; Atkinson et al., 2019). Identifying selective pressures and the resulting causal factors between non-linguistic aspects, such as the environment, culture, and social network dynamics, may highlight how and why linguistic structures change through time in light of the actuation problem (Weinreich et al., 1968; Yu, 2023). That is, why does a particular change happen in some language with some set of features, but not in another language with the same feature constellation? This was one of four problems posed to historical linguists by Weinreich et al. (1968) with the aim to investigate how and why languages change, and it still remains a null model from which to test linguistic vs. non-linguistic pressures.

In recent years, increasing evidence suggests that languages adapt to various external pressures at different levels of communication and lingual propagation, e.g., through individual physiological changes (Moisik and Dediu, 2017; Blasi et al., 2019; Everett and Chen, 2021), speaker accommodation strategies (Lindblom, 2000; Roberts and Clark), and global factors resulting in widespread patterns of correlation between non-linguistic factors and linguistic features (Bentz et al., 2018, Wichmann). Linguistic adaptation of lexical phenomena has long been studied (Sapir, 1912) and is clearly evident across cultures, e.g., differentiating lexically between “ice” and “snow” is more common in colder climates than warmer ones (Regier et al., 2016). But the evolution of words and their senses is more nuanced, e.g., the colexification of “cloud” and “fog” in the Andean highlands is language-family specific (Urban). This line of research highlights some of the challenges and biases in making observations of global trends of linguistic phenomena. Another interesting challenge is *blue*. An environmental factor affecting color vocabulary across languages worldwide is argued to be due to lens brunescence of speakers in regions with high rates of ultraviolet light (particularly UV-B); resulting over time in less visible light in the blue area of the spectrum, and thus decreased words for “blue” across these languages (Dediu). But rather than perceptual salience, Gibson et al. (2017) argue that color names reflect color use across cultures due to communicative needs (it has also been shown that speakers of languages with more than one term for blue react faster in color perception tests, see Winawer et al., 2007). Finally, another striking area of investigation is the conventionalization of spatial conception, which differs across cultures and languages (Levinson, 1998); recent experiments show that the use of spatial terms is environmentally adaptive in virtual reality contexts (Nölle et al., 2020). These studies and many others raise the important questions of how non-linguistic factors affect our species cognitively, culturally, and linguistically, ultimately impacting on the lexicons of the world's languages.

In research over the last few years, it appears that phonetic, phonological, morphological, and syntactic features of languages seem to change, or adapt to, non-linguistic pressures. For example, languages spoken by large populations reduce the complexity of their inflectional morphology, suggesting that grammar that is difficult for adult learners to acquire is less likely to be transmitted to future generations (Lupyan and Dale, 2010). Similarly, it has been shown that languages with more second language learners tend to lose nominal case, also suggesting that adult learners reduce morphological complexity in situations of high degrees of language contact (Bentz and Winter, 2013). Likewise, languages from larger families resulting from demographic spread have been found to be associated with obligatory marking of TAM (Tense-Aspect-Mood) marking (Gil, 2021), whereas increased sociopolitical complexity seems to correlate with increased grammaticalization of thematic-role assignment (Gil and Shen, 2019). Some linguists (e.g., McWhorter, 2001; Parkvall, 2008) have suggested that creoles, with their notable structural simplicity, particularly their extreme morphological simplification, would represent an extreme instance of these effects (see Good, 2012 or Mufwene, 2013 for more nuanced views). Overall, these findings support the argument that languages are adaptive systems, and in particular, the effects can be observed when large non-native speaker populations come into intense language contact situations (Bentz et al., 2015). Measuring linguistic complexity, of course, comes with many challenges, and so far there are few, if any, agreed upon methods for its operationalization (Ansaldo and Nordhoff, 2009; Sinnemäki, 2011; Moran and Blasi, 2014; Newmeyer and Preston, 2014; Ehret et al., 2021; Bentz et al., 2022; Benítez-Burraco et al.).

Most productive and successful research has investigated adaptive changes in spoken phonetics and phonology because of data access. Speculations go back centuries. Recent work, however, highlights the importance of moving beyond “simple” observations or correlation analyses by taking into account statistical confounds due to phylogenetic and spatial autocorrelation. For example, Hay and Bauer (2007) cautiously reported a significant correlation between phoneme inventory size and language population size. But **?** showed that once genealogical relatedness (phylogenetic bias) is taken into account as a confounding factor (e.g., using hierarchical linear mixed models), there is no correlation (cf. Cysouw et al., 2012). More recent research has expanded this approach by incorporating not only language family bias, but also linguistic areas as confounding factors into statistical models (Guzmán Naranjo and Becker, 2022; Hartmann, 2022; Hartmann et al., 2024). This is in light of the fact that dealing with statistical biases in linguistics data is a difficult and unsolved problem. Since at least the 1970s, language scientists have been confronted with phylogenetic relatedness, language contact, and the actuation question (i.e., why does a particular linguistic change happen in one language, but not another one with the same or similar situation and linguistic system?). No agreed upon methods have been adopted. Thus, proper statistical sampling and data quality have been perennial issues (Sherman, 1975; Bell, 1978; see discussion in Moran, 2019), but now datasets continue to be created and expanded at breathtaking rates for researchers, e.g., studying phonetic typology from thousands of hours of time-aligned and annotated recordings of thousands of speakers from dozens of typologically diverse languages (Ahn and Chodroff, 2022); or from millions of audio recordings of comparable speech tasks across nearly 1,000 language varieties across China (Liang et al., 2023). At present, things are not different in other domains of languages, such as grammar, e.g., the recent release of the Grambank database which covers 2,467 language varieties (from 215 different language families, as well as 101 isolates) and 195 grammatical features (Skirgård et al., 2023). While researchers still try to overcome the challenges that cross-linguistic language data has always presented, technological advances and increased data access brings with it new and interesting problems for analysis and causal inference (e.g., Moran et al., 2021; Maddieson, 2023; Hartmann et al., 2024).

Although in practice many researchers do not draw a clear distinction between statistical tests and causality, it should be clear that correlation and statistical inference do not mean causality within cross-linguistic or cross-cultural datasets (Bromham and Yaxley, 2023, inter alia). Regardless, recent studies investigating phonological diversity and non-linguistic factors bring together multiple lines of research to investigate, and thus try to validate, correlation patterns in terms of causality (Bromham and Yaxley, 2023; cf. Hernan and Robins, 2020). For example, empirical evidence suggests that adaptations of phonological systems are due to population differences in anatomy (Moisik and Dediu, 2017; Blasi et al., 2019; Dediu et al., 2019; Everett and Chen, 2021). Together with cross-linguistic observations, statistical analyses, and biomechanical modeling of the vocal tract and its movements, these models converge on the same idea that certain anatomical configurations result in decreased articulatory effort in speech sound production, and thus likely create a measurable diachronic signal in and across phonological systems (Liljencrants and Lindblom, 1972). The idea that phonological repertoires evolve due to external (e.g., environmental) pressures basically boils down to the principle of least effort (Hartmann et al., 2024). That is, vocal tracts are adapted for minimizing biomechanical effort and linguistic systems for increased communicative efficiency (Levshina and Moran, 2021). Things can be expected not to be different with regard to other domains of language and other dimensions of human physiology. For instance, whereas some aspects of morphology and syntax are rule-dependent (like verbal inflection or word order), some others appear as idiosyncratic (like suppletive forms or idioms). Cognitively, rules are stored in our procedural memory, whereas irregularities are stored, together with the lexicon, in our declarative memory (Ullman, 2015). One could hypothesize that a differential reliance of the world's languages on rule-dependent vs. rule-independent phenomena might result in a differential potentiation of these two types of memories in speakers; a more radical view would be, of course, that changes external to language impacting these memory systems could favor, or even trigger, the transition from one type of language to the other (see Chen et al., 2023 for discussion).

An area of ongoing debate that captures much of the heated back-and-forth regarding differing opinions and issues of statistical bias and data quality in comparative linguistics, is that of environmental factors of climate (Munroe et al., 1996; Fought et al., 2004; Everett, 2013; Roberts and Winters, 2013; Maddieson and Coupé, 2015; De Boer, 2016; Hammarström, 2016; Ladd, 2016; Moran, 2016; Maddieson, 2018; Roberts, 2018; Urban and Moran, 2021)—specifically the lack of humidity (aridity)—on the emergence of certain uses of the vocal cords (Everett, 2013, 2017; Everett et al., 2015, 2016; Maddieson and Coupé, 2015; Maddieson, 2018; Hartmann, 2022; Hartmann et al., 2024). The basic idea is that over thousands of years, languages spoken in dry areas are less likely to rely on, e.g., complex lexical tonal contrasts, because of the impact of desiccation on larynx function (Everett et al., 2015, 2016; Everett, 2017). Empirical and experimental evidence clearly shows that the larynx is prone to desiccation within very short time frames, resulting in a negative effect of noise-to-harmonics ratio, including diminished voice quality as measured by jitter and shimmer rates (Alves et al., 2017). However, whether a sustained effect on speech production over hundreds or thousands of years has resulted in observable diachronic trends in phonological inventories depends on who, and how, one asks. Work by several researchers supports a desiccated environment and lack of complex tonal systems (Everett et al., 2015; Everett, 2017; Liang et al., 2023); whereas other researchers using different analytical approaches and/or data do not find a significant effect (e.g., Hammarström, 2016; Roberts, 2018; Hartmann, 2022; Hartmann et al., 2024).

This back-and-forth with no clear cut answer is indicative of the myriad factors involved in asking whether there are causal factors from non-linguistic pressures leading to language adaptation, and whether it is discernible through observable diachronic change. Recent research revisiting the issue of aridity and tonogenesis is undertaken by Liang et al. (2023), who examined the rates of jitter in over a million audio files recorded with similar stimuli, methods, and equipment, from nearly 1,000 different locations across China. Jitter is used as a proxy for measuring the imprecision of vocal fold vibration, such that higher figures of jitter are a cue of more inconsistent fundamental frequency. Their findings overall support the research by Everett et al. (2015), but the approaches put forth by Liang et al. (2023) have not gone without criticism. Hartmann et al. (2024) report that geospatial and historical autocorrelation were not controlled for, because climate changes through time (cf. Roberts, 2018; Gannon et al., 2023). So like languages, we cannot assume that the current state of things was always the same—in linguistics the inverse is known as the uniformitarian principle (Labov, 1972; Walkden, 2019), i.e., that the current distribution of features across languages are the same, similar, or at least useful, for predicting aspects of languages in the past.

This issue of temporal bias was raised by Moran et al. (2020) when comparing present day language data with that of ancient and reconstructed languages. Whereas, phylogenetic and spatial autocorrelation can be reasonably removed as confounds through statistical approaches, comparing languages—or other variables—through time is particularly problematic because reconstructions are not temporally homogeneous. For example, we cannot simply bin “old” vs. “modern day” languages and compare them (cf. Moran et al., 2021). Furthermore, language families are not homogeneous in their size or their branching, i.e., their diversity. Indo-European is a large language family with many branches, but Basque is, for all intensive purposes, a language family with one branch. Statistical inference on the two phylogenies is biased if we are comparing languages across different taxonomic levels, and across different language families (Moran et al., 2020).

Temporal bias—together with history—raises a crucial issue that must be addressed, i.e., what is the recent impact of colonalization on the world's languages? The little research in this area suggests a homogenizing process, at least in phonetics, observable in the diachronic signals in global linguistic trends of the recent past (Moran et al., 2020). For example, Blasi et al. (2019) conclude that post-Neolithic changes in bite configuration contributed to the widespread emergence of labiodentals. However, more recent research suggests their global spread is measurably very recent—in the last few hundred years or so—because colonizing languages, including Portuguese, Spanish, French, English, Russian, Arabic, and Indonesian, all largely had labiodentals in their languages before they came into contact with other speaker communities, who already had a sustained overjet/overbite configuration, making their adoption less biomechanically demanding. There is a statistically significant difference in the typological frequency of labiodentals, and other sound classes including affricates, between ancient and reconstructed languages, when taking temporal bias into account (Moran et al., 2021). These findings support the idea that the phonological inventories of present day languages have been clearly impacted in terms of their composition during the last few hundred years of colonialization. Thus, like the diachrony of language change through time, Hartmann et al. (2024) suggest that non-linguistic “historical” variables, such as climate which is known to change through time and has impacted the human body and behavior (Warden et al., 2017; Klein et al., 2023; Margari et al., 2023), be accounted for as a confound. That said, as one goes back to a deeper past, this homogeneity can be safely expected to be replaced by real discontinuities. For instance, Benítez-Burraco and Progovac (2020) have hypothesized that humans might have spoken simpler languages (both morphologically and syntactically) perhaps as late as 50,000 years ago, because our less cooperative behavior made the complexification of languages more difficult through cultural mechanisms.

Overall, researchers must be aware of the potential effects of the nature of past and recent population contact situations, and their dynamics, as well as the dynamics of the non-linguistic variables under focus. Ideally, work in genetics in these regards will also shed further light on the problems, and solutions, involved in studying language change throughout time, since genetics enables us to reconstruct detailed human genealogies and populations movements in the past (Sikora et al., 2017; Skoglund and Mathieson, 2018; Bose et al., 2021; Ning et al., 2021; Serrano et al., 2021; Barbieri et al., 2022). For instance, family pedigrees and mating practices can be confidently inferred from ancient DNA and later used to estimate the nature of social networks, which together with other factors, like population number or forms of sociopolitical organization, seem to play a key role in shaping language features, as discussed above. Likewise, patterns of gene diffusion and genetic structuring, as inferred from present-day populations, but also from ancient DNA, can help gain a good knowledge of population displacements and admixtures in the past, which are known to fuel language change through language split and divergence, and language contact, respectively.

In sum, investigating language evolution requires “new” methods for studying causal associations between linguistic and non-linguistic variables. While researchers strive for methods for dealing with statistical biases including phylogenetic, spatial, and diachronic autocorrelation (Moran et al., 2021; Bromham and Yaxley, 2023), the current state of the art uses multifaceted strains of correlational evidence to try to support causality (Maddieson and Benedict). Additional experimental findings, “big” data, and new approaches to estimate causal effects from observational data, i.e., causal inference or causal networks (Roberts et al., 2020), are the current avenues aimed at fruitful progress. Finally, truly multidisciplinary research aimed to integrate different narratives of human evolution and human history will enable us to circumvent some of the problems and limitations discussed here.

In this second volume, we bring together 9 contributions from 22 scholars. These articles represent a breadth of investigations that investigate effects on the lexicons of languages (Urban; Khalilia et al.; Wichmann; Dediu), demonstrating environmental pressures on phonological systems (Maddieson and Benedict), revisiting the issue of complexity trade-off in linguistic subsystems (Benítez-Burraco et al.), and the emergence of linguistic features and systems (Roberts and Clark; Irurtzun), and finally how speech affects the physical environment (Everett et al.), instead of the other way around.

With regards to the lexicon, Urban's contribution investigates the adaptation of widespread colexification at high altitudes of the words for “fog” and “cloud”. While there is global support for this observation, Urban finds that the languages in the Central Andes paint a more nuanced picture. That is, by investigating colexification in Quechuan language family, whose speakers live at both low and high altitudes, Urban finds no support for adaptive processes within language families. This suggests that there are lineage-specific preferences for and against colexification, which supports previous claims that, for example, report differential rates of lexical change per language family with population size potentially playing a role (Greenhill et al., 2018) or that phonological systems exhibit differential rates of change in lineage-specific ways (Moran and Verkerk, 2018).

Concerning population size, Wichmann's original research article builds on previous reports that there is an inverse relationship between population size and word length, additionally showing that languages are more likely to have contrastive lexical tone when they have shorter words. Wichmann therefore hypothesizes that the causal relationship between population size and a decrease in mean word length leads to the increased probability of languages having tone or an increase in their number of tones. This causal relationship is reportedly most prominent in Subsaharan Africa and Southeast Asia, two areas known to have had large prehistoric populations that blossomed during the Neolithic revolutions, probably related to the adoption of agriculture (Bellwood, 2004). In this sense, Wichmann argues that tone would have been much less frequent in the world's languages in pre-Neolithic times (see also Maddieson, 2023).

Chen et al. (2023) report that close-knit small population societies with limited contacts culturally tend to have languages with more complex morphologies. This idea goes back to at least Trudgill (1989, 1996, 1998). Although it has been suggested that complexity trade-offs between morphology and syntax may have been inhibited by the advent of writing (e.g., Karlsson, 2009), Benítez-Burraco et al. in this Research Topic find a positive correlation between complexity of morphology and syntax, instead of a negative or “equally complex” trade-off. Again, the findings seem to be language family specific, and are ultimately driven by certain language families. It is an ongoing research question, i.e., within domains of linguistic complexity, what external factors on languages shape and mold linguistic structures?

In this vein, Roberts and Clark's original research article explores the emergence of phonological structure by investigating how interlocutors approach a communication task. They find that phonological dispersion appears when small-scale choices and adjustments lead to large-scale consequences and structures. This study is concerned in detail on how phonological systems organize themselves, in light of what we know from decades of research on how phonological inventories are organized and how they tend to follow patterns of symmetry, and in the vowel system in particular, dispersion to the cardinal vowels (Liljencrants and Lindblom, 1972; Stevens, 1989; Schwartz et al., 1997; de Boer, 2000).

Maddieson and Benedict's research is concerned with demonstrating environmental impacts on the phonological structure of languages, which has a long history as we have also noted above. As they point out, there are myriad ways to collect, curate, and analyze data from very different sources, including phonological information encoded in grammars, information about where languages are spoken, and environmental data provided from several different sources and at different resolutions and time depths. As such, the authors highlight for example the problems with temperature records and language locations. Their results suggest that some of the previously proposed environmental impacts on languages are statistically valid, but these findings need to be investigated in terms of a broader framework of language types, and ultimately factors involved in language relatedness and areal contact. Theirs is a cogent case study on many aspects relevant to the study of non-linguistic factors affecting language adaption, including issues of language sampling, language locations (points are most often used, instead of polygons), statistical bias in controlling for inheritance and areality, and proper statistical hypothesis testing. This original research article provides a blueprint for future studies investigating climatic variables and their potential influence on phonological systems of the world's languages.

Returning to potential evolutionary and cultural pressures on the lexicon, the study by Khalilia et al. looks at lexical diversity in kinships across dialects and languages. As noted, it has long been known that the environment plays a role in the lexicons of languages. Kinship systems have also long been known to divide up the worldview in diverse ways (Morgan, 1871). Kinship systems and their vocabulary have been typologized in standardized ways, with many particular patterns found across the globe, and others known to be extremely rare (e.g., see Mansfield, 2013). Khalilia et al. have created a browsable and downloadable computational resource for investigating kinship terminology systems from a large sample of languages, from which they undertake two case studies on Arabic dialects and three Indonesian languages. This work provides not only data for other researchers, but insights into the diversity of kinships and the drivers of their diversity.

Another interesting proposal of environmental factors on lexical diversity is studied by Dediu, in his research article on ultraviolet light effects on color vocabulary. Using a large language sample (*N* = 834), Dediu investigates whether speakers living in regions with high levels of UV-B and whether those languages are more or less likely to have a term for “blue”. The causality here is suggested as people living in areas of high ultraviolet light (e.g., around the tropics) are more prone to develop lens brunescence (think cataracts), which ultimately affects the perception of visible light in the blue spectrum. It is recently well-studied that color perception is to some extent individual-specific—recall “The Dress” episode a few years back that had the internet divided between whether the dress was blue and black, or white and gold.^1^,^2^ This dichotomy of opinions led to an incredible amount of new research on color perception and linguistic relativity more broadly. Building on previous research with a larger language sample that allows him to address issues of phylogenetic autocorrelation, Dediu finds strong support that the color lexicons of languages in areas of high ultraviolet light are less likely to have a term for “blue”, which he argues is amplified through time by language use and transmission.

In terms of various factors leading to the emergence of linguistic structure, the opinion piece by Irurtzun investigates how biology, culture, and environment impact the emergence of (alternate) sign languages. Irurtzun argues that language modality can be determined by these factors, i.e., that the design of “new” languages is independent of emergent diachronic pressure from local and oral language structures. Irurtzun's opinion piece provides evidence and argumentation against *a priori* language external factors affecting the emergence of core aspects of language, including grammar and phonology. All in all, it is argued that non-linguistic pressures affect language design, and in this case, environmental, cultural, and biological factors affect the choice of modality of language production.

Lastly, the research article by Everett et al. takes the idea of non-linguistic pressures on language structure and flips it on its head. The authors ask how speech affects the physical environment. Since COVID-19, there has been increased interest in aerosol production and disease transmission, with at least one old study (Inouye, 2003) being revisited, and newer studies also speculating that different languages' phonologies transmit aerosols in greater or lesser amounts (Asadi et al., 2019, 2020; Hamner, 2020; Stadnytskyi et al., 2020; Bahl et al., 2021). Although this line of research was quick to be criticized, detailed empirical and experimental evidence has been lacking. Thus, Everett et al. create new methods for measuring aerosol production; itself a complicated thing to measure because aerosols from the throat and/or lungs vary greatly in microscopic sizes. Their novel approach and combination of various physical machinery (e.g., pneumotachograph, electrical particle impactor) allow the authors to attain physical resolutions not yet measured in the previous literature, allowing for well-described effects of aerosols from different speech sound classes. Although most of us would prefer to forget about COVID-19, the research approach and agenda presented by Everett et al., allows researchers to analyze and discuss how speech sounds generate aerosol emissions that are relevant to airborne disease transmission in the physical environment.

## Author contributions

AB-B: Conceptualization, Funding acquisition, Investigation, Methodology, Validation, Writing – original draft, Writing – review & editing. SM: Conceptualization, Funding acquisition, Investigation, Methodology, Validation, Writing – original draft, Writing – review & editing.

## Footnotes

^1^ https://blogs.scientificamerican.com/illusion-chasers/the-science-ball-where-everybody-wore-the-same-dress/

^2^ https://blogs.scientificamerican.com/illusion-chasers/the-current-biology-of-the-dress/
